# Towards control of cellulose biosynthesis by *Komagataeibacter* using systems-level and strain engineering strategies: current progress and perspectives

**DOI:** 10.1007/s00253-020-10671-3

**Published:** 2020-06-11

**Authors:** Małgorzata Ryngajłło, Marzena Jędrzejczak-Krzepkowska, Katarzyna Kubiak, Karolina Ludwicka, Stanisław Bielecki

**Affiliations:** Institute of Molecular and Industrial Biotechnology, Lodz University of Technology, B. Stefanowskiego 4/10, 90-924 Lodz, Poland

**Keywords:** *Komagataeibacter*, Bacterial nanocellulose, Systems biology, Strain engineering, Molecular physiology, Composites

## Abstract

The strains of the *Komagataeibacter* genus have been shown to be the most efficient bacterial nanocellulose producers. Although exploited for many decades, the studies of these species focused mainly on the optimisation of cellulose synthesis process through modification of culturing conditions in the industrially relevant settings. Molecular physiology of *Komagataeibacter* was poorly understood and only a few studies explored genetic engineering as a strategy for strain improvement. Only since recently the systemic information of the *Komagataeibacter* species has been accumulating in the form of omics datasets representing sequenced genomes, transcriptomes, proteomes and metabolomes. Genetic analyses of the mutants generated in the untargeted strain modification studies have drawn attention to other important proteins, beyond those of the core catalytic machinery of the cellulose synthase complex. Recently, modern molecular and synthetic biology tools have been developed which showed the potential for improving targeted strain engineering. Taking the advantage of the gathered knowledge should allow for better understanding of the genotype–phenotype relationship which is necessary for robust modelling of metabolism as well as selection and testing of new molecular engineering targets. In this review, we discuss the current progress in the area of *Komagataeibacter* systems biology and its impact on the research aimed at scaled-up cellulose synthesis as well as BNC functionalisation.

**Table:** 

**Key points** • *The accumulated omics datasets advanced the systemic understanding of Komagataeibacter physiology at the molecular level.* • *Untargeted and targeted strain modification approaches have been applied to improve nanocellulose yield and properties.* • *The development of modern molecular and synthetic biology tools presents a potential for enhancing targeted strain engineering.* • *The accumulating omic information should improve modelling of Komagataeibacter’s metabolism as well as selection and testing of new molecular engineering targets.*

**Key points**

• *The accumulated omics datasets advanced the systemic understanding of Komagataeibacter physiology at the molecular level.*

• *Untargeted and targeted strain modification approaches have been applied to improve nanocellulose yield and properties.*

• *The development of modern molecular and synthetic biology tools presents a potential for enhancing targeted strain engineering.*

• *The accumulating omic information should improve modelling of Komagataeibacter’s metabolism as well as selection and testing of new molecular engineering targets.*

## Introduction

Bacterial nanocellulose (bionanocellulose, BNC), composed of β-1-4-linked glucose units, is a natural biopolymer produced by a wide variety of bacteria. Dimensions of BNC fibres give this polymer properties of a nanomaterial. Due to its unique features, BNC has found applications in various areas of biotechnology, medicine and industry (reviewed in Jang et al. 2017; García and Prieto 2019; Gorgieva and Trček 2019; Wang et al. 2019). It also constitutes a promising material in food applications, e.g. as rheology modifier, fat replacer, or in the immobilisation of probiotics and enzymes (reviewed in Azeredo et al. 2019). Despite many valuable features and a wide array of applications, products made of BNC are not yet present in our everyday life. This is because BNC production performance is still relatively low since its large-scale industrial fermentation process is more complex and highly capital intensive, especially in comparison with plant cellulose–based processes and pulp and paper industry. Therefore, much of an effort has been devoted to design an economical process for BNC production by optimisation of culture medium composition, cultivation mode and process parameters, bacterial strain, as well as postproduction purification (Krystynowicz et al. 2002; Jozala et al. 2016; Islam et al. 2017). Further improvements in these areas are necessary for increasing BNC production performance and making it more cost-effective.

The most efficient bacterial cellulose producers are acetic acid bacteria (AAB) of the *Komagataeibacter* genus, which synthesise high amounts of this biopolymer from a wide variety of carbon and nitrogen sources (Islam et al. 2017). *K. xylinus* is the bacterium in which the molecular mechanism of BNC synthesis was first revealed and studied. The biochemical pathway and the core catalytic machinery responsible for cellulose synthesis have been delineated (Ross et al. 1991; Saxena et al. 1994; Römling 2002; Velasco-Bedrán and López-Isunza 2007; Jacek et al. 2019a). Polymerisation of UDP-glucose into cellulose is catalysed by the bacterial cellulose synthase (BCS) enzyme complex. Two types of cellulose synthase operons are encoded by the *Komagataeibacter* genomes, i.e. type I and type II (Umeda et al. 1999). The type I cellulose synthase operon (*bcsI*) comprises four genes, *bcsAI*, *bcsBI*, *bcsCI* and *bcsDI*. The *bcsI* operon is flanked by accessory genes (*cmcAx*, *ccpAx* and *bglAx*), which modulate the process of cellulose synthesis (Römling and Galperin 2015; Jedrzejczak-Krzepkowska et al. 2016). The type II operon (*bcsII*) is probably responsible for the production of acylated cellulose due to the presence of an acyltransferase gene within its structure (Umeda et al. 1999). Expression of the cellulose biosynthesis operon was found to be constitutive, however shown to fluctuate depending on growth phase and environmental conditions (Augimeri and Strap 2015; Hernández-Arriaga et al. 2019; Ryngajłło et al. 2019a). Cellulose synthase enzyme activation occurs at the posttranslational level and is mediated by c-di-GMP (*bis*-(3′,5′)-cyclic di-guanosine-mono-phosphate), which binds to the PilZ domain of the BcsA subunit and activates it allosterically (Ross et al. 1985; Fujiwara et al. 2013; Römling et al. 2013). The cellular level of c-di-GMP is controlled by diguanylate cyclases (DGCs) and c-di-GMP-specific phosphodiesterases (PDEs) (Tal et al. 1998; Römling 2012).

The development of high-throughput omics techniques created an opportunity to study the *Komagataeibacter* species as systems and to venture beyond the core catabolic machinery of cellulose synthase. In this review, we discuss the current progress in the systemic understanding of *Komagataeibacter* physiology at the molecular level. We review the high-throughput studies which provided a global overview of different functional layers of a *Komagataeibacter* cell. The examples of untargeted and targeted approaches as well as genetic engineering strategies for strain improvement in terms of BNC synthesis intensification and features modification are also further presented.

### Systems-level perspective on the physiology of the *Komagataeibacter* species

Bacteria of the *Komagataeibacter* genus are considered as the model species for studying cellulose biosynthesis process. However, it cannot be regarded as a model species for molecular analysis since systems biology of *Komagataeibacter* is a relatively young research field, when compared with other model bacteria species, such as *Escherichia coli*, *Corynebacterium* sp., *Bacillus* sp. or *Pseudomonas* sp. Over the past decade, the first complete genome sequences became available for this genus and information of other omes started to accumulate. Application of the modern high-throughput omics techniques should enable obtaining of more of the systems-level information for the *Komagataeibacter* spp. and rapidly advance them to truly ‘model species’ status for studying molecular aspects of BNC synthesis.

### The sequenced *Komagataeibacter* genomes

The first full *Komagataeibacter* genome representation deposited in the GenBank database was for *K. hansenii* ATCC 23769 in 2010 (Iyer et al. 2010). Sequencing of the *Komagataeibacter* genomes accelerated with the appearance of the next-generation sequencing technology, and currently there are 54 genome sequences available (accession date March 2020, NCBI). The majority of these genomes were assembled based on short reads originating from such technologies as Illumina and consists of multiple (usually, several hundreds) of contigs. However, it is difficult to obtain an assembly of a high quality, i.e. low number of contigs, using this approach, since these genomes were shown to contain a large number of insertion sequences (IS), which often contribute to contig breaks during assembling (Coucheron 1993; Ryngajłło et al. 2019b). The low-quality genomes can be used for taxonomical analysis; however, they are of a limited use for the analyses which strongly depend on the integrity of annotations, such as comparative genomics (Ryngajłło et al. 2019b). Fortunately, the *Komagataeibacter* genome collection is becoming enriched in complete genome sequences such as those generated using PacBio SMRT or a combination of Illumina and Oxford Nanopore MinION sequencing technologies.

**Fig. 1:**
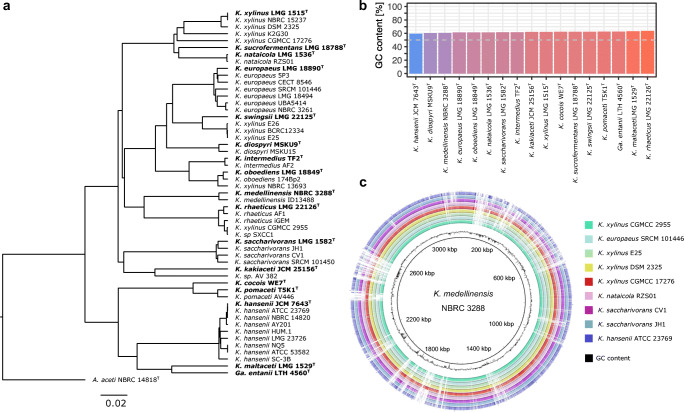
Sequence similarity of the *Komagataeibacter* genomes. **a** Phylogenetic tree based on ANI analysis performed using PYANI (0.2.9) python program employing BLAST+ program (Camacho et al. 2009; Pritchard et al. 2016). The UPGM tree based on ANI − 1 values was calculated using phangorn R package (Schliep 2011). The *Acetobacter aceti* 14818 was used as an outgroup. The tree was drawn in FigTree program (v.1.4.4). The scale bar represents the sequence divergence. **b** GC content of the type *Komagataeibacter* genomes. **c** Alignment of the closed chromosomal sequences of the *Komagataeibacter* genomes using BRIG (Alikhan et al. 2011)

The majority of the type strains of the *Komagataeibacter* species have been recently sequenced (Škraban et al. 2018) (Table 1). In total, the NCBI genome assembly collection represents 16 *Komagataeibacter* species: *K. xylinus*, *K. sucrofermentans*, *K. nataicola*, *K. europaeus*, *K. swingsii*, *K. diospyri*, *K. intermedius*, *K. oboediens*, *K. medellinensis*, *K. rhaeticus*, *K. saccharivorans*, *K. kakiaceti*, *K. cocois*, *K. pomaceti*, *K. hansenii* and *K. maltaceti* (Table 1). In the NCBI database, there is also deposited the genome of a *Gluconacetobacter entanii* LTH 4560 strain which was not reclassified to the *Komagataeibacter* genus since it is no longer available at the strain repository. The availability of the type strain genome sequences greatly aids phylogenetic classification of a new cellulose-producing isolate based on the whole-genome alignment measures such as, e.g., Average Nucleotide Identity (ANI) values (Goris et al. 2007; Richter and Rosselló-Móra 2009) or MUM index (MUMi; Deloger et al. 2009; Ryngajłło et al. 2019b). Based on the whole-genome comparison, it can be noticed that some of the sequenced *Komagataeibacter* strains were misclassified (Fig. 1a). These are mostly the various *K. xylinus* species, which do not form a clade with the *K. xylinus* LMG 1515 type strain. This is mainly because, formerly, the diversity of the cellulose producing AAB was underestimated and it was common to call a ‘*xylinus*’ strain any new isolate. What is characteristic of all the *Komagataeibacter* genomes is the high % GC content which is the highest for the *K. rhaeticus* LMG 22126 (63.5 %) and the lowest for the *K. hansenii* JCM 7643 (59.3%) (Fig. 1b). There are currently 11 genomes with closed chromosomal sequences (Fig. 1c). Of these, eight are complete genome assemblies consisting of one chromosome and several plasmids (Table 2); three genomes have been assembled at the chromosome level only (*K. hansenii* ATCC 23769, *K. rhaeticus* iGEM, *K. xylinus* CGMCC 2955). Based on the complete genome assemblies, the average number of genes encoded by a *Komagataeibacter* genome is 3571, which include 3327 protein coding genes. Comparative genomics analysis employing 19 complete and draft sequences has predicted the core genome constituting 1578 orthologous gene clusters (present in every *Komagataeibacter* genome; Ryngajłło et al. 2019b). A similar analysis involving only seven complete *Komagataeibacter* genomes has found 1719 core genes which make up 21.9% of a non-redundant set of all genes in these genomes (Jang et al. 2019). These results suggest that the *Komagataeibacter* species have more similar genome contents and share a greater portion of the core genes than other notable groups of bacterial species, such as *Mycoplasma* or *Bifidobacterium* (Jang et al. 2019).

**Table 1:** Characteristics of *Komagataeibacter* type strains and their genome sequences

Type strain	RefSeq assembly accession	Total genome size (Mb)	GC (%)	Assembly level	Isolation source	Cellulose synthesis?	Number of sequenced strains	References
*K. xylinus* LMG 1515	GCF_003207795.1	3.66	62.22	Contig	Mountain ash berry	+	5	(Gosselé et al. 1983; Yamada et al. 2012)
*K. sucrofermentans* LMG 18788	GCF_003207865.1	3.36	62.33	Contig	Black cherry, Japan	+	1	(Toyosaki et al. 1995; Yamada et al. 2012)
*K. nataicola* LMG 1536	GCF_003207795.1	3.67	61.48	Contig	Nata de coco, Philippines	+	2	(Lisdiyanti et al. 2006; Yamada et al. 2012)
*K. europaeus* LMG 18890	GCF_000285295.1	4.23	61.26	Scaffold	A submerged culture vinegar generator, Germany	−	7	(Sievers et al. 1992; Yamada et al. 2012)
*K. swingsii* LMG 22125	GCF_003207895.1	3.73	62.40	Contig	Apple juice, Italy	+	1	(Dellaglio et al. 2005; Yamada et al. 2012)
*K. diospyri* MSKU9	GCF_006538165.1	3.76	60.43	Contig	Persimmon, Thailand	+	2	(Naloka et al. 2019)
*K. intermedius* TF2	GCF_000964425.1	3.88	61.60	Scaffold	Kombucha beverage, Switzerland	+	2	(Boesch et al. 1998; Yamada et al. 2012)
*K. oboediens* LMG 18849	GCF_003207815.1	3.78	61.35	Contig	Submerged red wine vinegar, Germany	−	2	(Sokollek et al. 1998; Yamada et al. 2012)
*K. medellinensis* NBRC 3288	GCF_000182745.2	3.51	60.58	Complete genome	Vinegar, Japan	−	2	(Castro et al. 2013)
*K. rhaeticus* LMG 22126	GCF_003207855.1	3.47	63.49	Contig	Apple juice, Italy	+	3	(Dellaglio et al. 2005; Yamada et al. 2012)
*K. saccharivorans* LMG 1582	GCF_003207825.1	3.35	61.59	Contig	Beet juice, Germany	−	4	(Lisdiyanti et al. 2006; Yamada et al. 2012)
*K. kakiaceti* JCM 25156	GCF_000613305.1	3.13	62.14	Contig	Kaki vinegar, Japan	+	1	(Iino et al. 2012)
*K. cocois* WE7	GCF_003311635.1	3.41	62.27	Scaffold	Contaminated coconut milk, China	+	1	(Liu et al. 2018a)
*K. pomaceti* T5K1	GCF_003207955.1	3.45	62.53	Contig	Apple cider vinegar, Slovenia	+	2	(Škraban et al. 2018)
*K. hansenii* JCM 7643	GCF_000964405.1	3.71	59.28	Scaffold	Vinegar, Israel	−	9	(Gosselé et al. 1983; Yamada et al. 2012)
*K. maltaceti* LMG 1529	GCF_003206475.1	3.64	63.18	Contig	Malt vinegar brewery acetifier	−	1	(Slapšak et al. 2013)
*Ga. entanii* LTH 4560	GCF_003206495.1	3.60	62.57	Contig	High-acid spirit industrial vinegar, Germany	−	1	(Schüller et al. 2000; Yamada et al. 2012)

+, cellulose synthesis

**Table 2:** General properties of the complete *Komagataeibacter* genomes deposited at the NCBI database

Strain	RefSeq assembly accession	Number of chromosome sequences	Number of plasmid sequences	Chromosome size (Mb)	Plasmid sizes (kbp)	Protein coding genes	Pseudogenes	rRNA	tRNA	Other RNA
*K. xylinus* E25	GCF_000550765.1	1	5	3.45	336.14; 87.18; 26.30; 5.53; 2.22	3408	224	15	57	4
*K. xylinus* CGMCC 17276	GCF_009834365.1	1	3	3.53	233.86; 190.75; 31.01	3599	170	15	57	4
*K. xylinus* DSM 2325	GCF_004006375.1	1	2	3.35	368.64; 5.31	3330	182	13	56	4
*K. nataicola* RZS01	GCF_002009295.1	1	6	3.49	102.28; 39.91; 38.68; 37.91; 30.55; 25.77	3351	168	15	59	4
*K. europaeus* SRCM 101446	GCF_002173515.1	1	3	3.45	309.05; 37.20; 2.90	3294	138	15	58	4
*K. medellinensis* NBRC 3288	GCF_000182745.2	1	7	3.14	255.87; 76.07; 28.57; 4.78; 4.62; 4.26; 2.22	3108	195	15	57	4
*K. saccharivorans* CV1	GCF_003546645.1	1	6	3.19	217.54; 159.31; 127.84; 42.58; 26.65; 1.87	3277	144	15	57	4
*K. saccharivorans* JH1	GCF_004348195.1	1	14	3.10	221.73; 183.53; 92.69; 59.56; 41.06; 5.32; 4.90; 3.05; 2.81; 2.29; 2.00; 1.46; 1.09; 0.53	3251	123	15	57	4

Various phylogenetic analyses based on the *Komagataeibacter* genomes have highlighted a strong separation of the *K. hansenii* strains from the other species (Škraban et al. 2018; Jang et al. 2019; Ryngajłło et al. 2019b). Several of the predicted functional features of the *Komagataeibacter* genomes followed this phylogenetic separation. It has been shown that the structure of the first cellulose synthase operon (*bcsI*) differs between these two groups, whereas in the *K. hansenii* genomes it has been shown that the *bcsA* and *bcsB* genes are fused (Saxena et al. 1994; Matsutani et al. 2015; Ryngajłło et al. 2019b). Moreover, the presence of the acetan biosynthesis gene cluster, which consists of 17 genes, was predicted in the majority of the *Komagataeibacter* genomes (Ryngajłło et al. 2019b). However, in the *K. hansenii* spp., the acetan cluster was absent, which suggested that they do not produce this exopolysaccharide (EPS) or produce one of a different composition. Moreover, it has been shown that the number of genes encoding enzymes metabolising c-di-GMP, the allosteric activator of cellulose synthase subunit A (*bcsA*), is much lower in the *K. hansenii* spp., which suggested that the c-di-GMP-based regulatory network is much less expanded in these species. What is more, it has been discovered that the presence of the CRISPR-Cas gene cluster is one of the distinctive features of the *K. hansenii* spp.

Further comparative genomics studies should benefit from the clarified taxonomy of the *Komagataeibacter* species. Recruitment of the genomic information and revisiting of the large number of physiological reports published in the past may highlight more common as well as distinctive functional features which characterise these species and explain the difference in BNC yield and structure.

### Global gene expression studies

While a genome sequence only gives information on the genetic content of a cell, studies involving investigation of gene expression patterns provide an insight and deepen the understanding of gene functions. The employment of RNA sequencing (RNA-seq) is particularly useful for gaining a global overview of dynamic changes at the transcriptional level. The first global transcriptome of a *Komagataeibacter* sp. has been published and involves the study of effect of ethanol supplementation in the culture of *K. xylinus* E25 (Kubiak et al. 2014; Ryngajłło et al. 2019a). This study has expanded the understanding of the positive effect of ethanol on BNC synthesis by revealing the gene expression changes triggering this effect, such as up-regulation of UDP-glucose synthesis pathway and down-regulation of glycolysis and acetan biosynthesis. Moreover, metabolic pathways responsible for ethanol metabolism were also delineated. Several of the previously not described genomic regions, such as acetoin metabolism and iron uptake, were also found to be important during the growth in ethanol-containing medium.

Recent availability of complete *Komagataeibacter* genomes should encourage more of RNA-seq-based transcriptomic studies which should give a better insight into regulatory pathways which are not well understood in the *Komagataeibacter* species. For example, up to 150 DNA-binding proteins are predicted in the *K. hansenii* ATCC 23769 genome (based on the predictions from MIST3 and P2RP database). One of the Crp/Fnr transcription factors was previously shown to be important for cellulose synthesis since it positively regulated the expression of the *bglAx* gene of the *bcsI* operon (Deng et al. 2013). However, apart from the Crp/Fnr, the gene targets of other transcription factors remain unknown. What is more, we do not know if the *bglAx* and the *bcsI* genes are the only components of the Crp/Fnr regulon. A chromatin Immunoprecipitation Sequencing (ChIP-Seq) study investigating Crp/Fnr DNA binding profile could clarify if its impact on global gene expression expands beyond the known gene targets.

Other RNA-seq approaches present a great potential for the analysis of the structure and dynamics of *Komagataeibacter* transcriptomes. For instance, sequencing of primary transcriptomes which involves enrichment of native transcripts bearing a 5′-triphosphate group, in another AAB *Gluconobacter oxydans* 621H, has shown to be useful for correction of start codons, prediction of operons as well as for identification of promoter motifs, 5′-untranslated regions (UTRs), ribosome binding sites (RBS) and *cis*-regulatory elements such as riboswitches (Kranz et al. 2018). Currently, the annotation of the *Komagataeibacter* genomes is being done mostly through automated prediction which is based on sequence homology and which could miss or incorrectly annotate genes and other functional genome elements.

### Proteomic studies

The measurement of cellular mRNA levels gives only partial information about gene expression since it also depends on post-transcriptional regulatory effects which influence mRNA lifetime. Identification and quantification of cellular protein content complements this picture and further improves understanding of the phenotype. A study involving *K. xylinus* E25 investigated and compared the protein profiles of wild-type (Cel+) and cellulose non-producing cells (cellulose-negative or Cel−, discussed later) using two-dimensional (2D) gel electrophoresis, peptide mass fingerprinting using MALDI-TOF mass spectrometry (MS) as well as by partial peptide sequencing with ion trap electrospray MS (Krystynowicz et al. 2005). This study revealed that the disappearance of cellulose-producing ability was related to the lack of two enzymes necessary for UDP-glucose synthesis: phosphoglucomutase and glucose-1-phosphate uridylyltransferase. Another study collated the protein profiles between a *K. xylinus* K3 strain isolated from Kombucha and its spontaneous mutant of lower cellulose productivity using 2D gel electrophoresis and MALDI-MS (Nguyen et al. 2010). Three out of five of the up-regulated protein spots in the mutant strain were selected and identified: chaperonin GroEL, short-chain dehydrogenase/reductase (SDR) and deoxythymidine diphosphate (dTDP)-4-dehydrorhamnose 3,5-epimerase. Specifically, the last protein was considered likely to be responsible for the reduced cellulose synthesis by the mutant strain due to its involvement in acetan biosynthesis and competition for the same carbon substrates as cellulose synthesis process.

A metaproteomics approach was undertaken to study the bacteria involved in the production of high-acid spirit vinegar using culture-independent method (Andrés-Barrao et al. 2016). Through 16S RNA and housekeeping genes (*dnaK*, *groEL*, *rpoB*) sequencing, it was found that the bacterial population of the culture was homogenous and belonged to unknown *Komagataeibacter* species which clustered closed to *K. hansenii* and *Ga. entanii* in a phylogenetic tree. The study analysed differentially expressed proteins between low- and high-acidity cultures using 2D-differential in-gel electrophoresis (2D-DIGE) and MS. Out of 2000 proteins detected, 32 most abundant and significantly up-regulated proteins from the high-acid condition were selected and identified. The majority of these proteins were involved in stress response, the TCA cycle and other metabolic processes.

The presented proteomic studies employ gel-based technologies, which are known to be more laborious and of limited scope and resolution. Application of more high-throughput approach by, e.g., coupling of liquid chromatography to MS (LC–MS), should allow for more efficient and broader applications.

### Metabolomic profiling

Metabolomics is commonly employed to give a global overview of changes in the metabolic status of an organism in response to environmental fluctuations, nutrition availability or genetic disturbances. While extracellular metabolites, such as gluconic or acetic acid, have been often measured in the cultures of the *Komagataeibacter* species, a recent study in *K. xylinus* CGMCC 2955 (*Gluconacetobacter* in the original work) investigated the differences between static and agitated cultures by measuring their internal metabolomic profiles using gas chromatography coupled with mass spectrometry (GC–MS) (Liu et al. 2015). In total, 79 intracellular metabolites were detected and quantified in both culture types. The results of multivariate data analysis showed clear separation of the samples from the tested cultures, what indicated that the cells in the static culture were metabolically distinct from those in the agitated one. It was shown that metabolites of the central carbon metabolism and amino acids biosynthesis pathways displayed significant changes in the samples from different culturing methods and sampling times. The metabolites responsible for distinguishing the samples were mainly trehalose, phosphate and gluconic acid. The agitated culture promoted cell growth and viability; however, the static culture was characterised by the highest conversion rate of glucose to BNC. Moreover, the initial accumulation of trehalose and amino acids (proline, glutamic acid, alanine, l-valine and l-threonine) in the agitated culture at the highest rotational speed was interpreted to fulfil a synergistic protective role during adaptation to the environmental stress caused by the culture condition, such as the hydrodynamic stress. At the molecular level, it has been shown that trehalose plays an important role in protein stabilisation by influencing the water environment surrounding a protein and promoting maintenance of their native state (Singer and Lindquist 1998; Ruhal et al. 2013; Olsson et al. 2016). In case of proline, its role in stress protection against various environmental factors is well documented and includes osmo- and thermo-protection, maintenance of protein stability and oxidative stress resistance (Christgen and Becker 2019). It has been shown in *E. coli* that proline oxidative metabolism generates hydrogen peroxide and thus increases oxidative stress tolerance via a preadaptive effect which involves induction of pathway for H_2_O_2_ removal and maintenance of redox homeostasis (Zhang et al. 2015). Due to the higher availability of oxygen in the agitated cultures of *K. xylinus* CGMCC 2955, degradation of glucose to gluconic acid proceeded very fast. The following rapid consumption of the gluconic acid in the cytosol may have resulted in generation of the oxidative stress in these cultures due to the increased electron flux through the respiratory chain.

It has been observed that the sufficient and uniform aeration of the agitated culture favours selection of cellulose non-producing cells, in comparison with the static culture (Park et al. 2003; Krystynowicz et al. 2005). The conclusion which can be drawn from the studies employing agitated cultures is that BNC production is a transient feature of the *Komagataeibacter* species and is maintained as long as it confers a survival advantage. Since these are obligate aerobic bacteria, formation of a cellulose pellicle in a liquid culture is advantageous since it enables the retention of cells close to the air–liquid interface where the concentration of oxygen is high and there is access to the nutrients from the medium.

More studies investigating exo- and intra-metabolic profiles under various conditions, relevant for cellulose biosynthesis, will definitely bring a valuable insight into the systemic understanding of a *Komagataeibacter* cell. Moreover, in the case of some of the metabolites, targeted approaches for isolation and quantification are needed. For the *Komagataeibacter* species, it is particularly important to study the profile of synthesised exopolysaccharides. Production of such EPS as acetan not only competes with the cellulose synthesis pathway for carbon precursors but was also shown to interfere with cellulose network structure, i.e. fibril assembly and crystallisation (Ishida et al. 2002; Fang and Catchmark 2014). The monosaccharide composition and yield of EPS synthesised has been found to vary depending on strain and carbon source (Fang and Catchmark 2015). Further research involving the impact of EPS synthesis by the *Komagataeibacter* species should contribute to engineering of BNC membranes of a custom structure and properties.

### Genome-scale metabolism modelling

Genome-scale metabolic models (GEMs), which employ both the genomic and biochemical information to describe gene–protein–reaction associations, have been shown to be particularly useful for systems-level metabolic studies (Gu et al. 2019). The recent availability of complete genome sequences of the *Komagataeibacter* strains stimulated reconstruction of GEMs to better understand their metabolism and to predict metabolic capabilities of the BNC producers. A GEM has been reconstructed for *K. nataicola* RZS01 and comprised 771 genes, 2014 reactions and 2035 metabolites (Zhang et al. 2017). Through constraint-based analysis, this model was used to characterise and evaluate cellular metabolism and to predict genes and reactions necessary for growth. The analysis revealed that glycerol is the most optimal carbon source for the highest BNC production. Moreover, the minimisation of metabolic adjustment (MOMA) algorithm was used to predict eight overexpression targets for increasing BNC production; however, these candidates were not experimentally tested. In the case of *K. hansenii* ATCC 23769, only the core metabolic model was reconstructed which included 74 reactions and 68 metabolites (de Souza et al. 2018). The metabolic flux distribution of this model was calculated using flux balance analysis (FBA) approach and in silico simulations were performed to predict the growing abilities on different substrates and the minimal medium capable of supporting BNC production. A more expanded GEM was constructed for *Komagataeibacter xylinus* DSM 2325 (Jang et al. 2019). This GEM was based on genome sequence and experimental growth data and contained information on 686 metabolic genes, 1810 reactions and 1712 metabolites. It was also used to predict gene overexpression targets for increased BNC production. Heterologous overexpression of two of these targets, glucose-6-phosphate isomerase (*pgi*) and phosphogluconate dehydrogenase (*gnd*) brought enhanced BNC production, which was 2-fold for the strain overexpressing the *E. coli pgi* gene. In another study of this team, exploiting this GEM and through simulated FBA, it was found that the inclusion of a reaction catalysed by phosphofructokinase (*pfkA*), which is absent in the *Komagataeibacter* spp., improves cellulose production rate and specific growth rate (discussed later; Gwon et al. 2019).

The functionality of GEMs greatly depends on the annotation status of a genome. Advancement in functional genomics of the *Komagataeibacter* species should improve GEMs and allow for generation of more precise models and the subsequent selection of a higher number of gene targets for further engineering.

### Untargeted and targeted approaches for improving BNC synthesis efficiency or properties

Two main approaches, untargeted and targeted, for modification of a cellulose producer have been employed. The untargeted methods usually involve induction of changes in culturing conditions or mutagenisation and require generation and testing of a large number of clones. Despite being laborious and time consuming, these approaches have successfully been employed to obtain producers of improved BNC yield. At the same time, the studies which were coupled with genetic analysis of the mutant strains enhanced the understanding of *Komagataeibacter* molecular physiology. On the other hand, rational strain design through targeted approaches is usually more efficient and may yield cellulose of specifically desired features. However, this approach would greatly benefit from the understanding of a whole system at a molecular level because, at present, cellular response to introduced changes is hardly predictable. We will discuss the studies employing both of these approaches for obtaining mutants producing BNC of improved yield and properties.

### Untargeted approaches

The early untargeted studies explored the natural phenotypic variability of some of the *Komagataeibacter* strains which often leads to spontaneous disappearance of cellulose-synthesising ability through the formation of cellulose non-producing cells (Table 3). It has been suggested that these phenotypic changes are induced due to the stress factors, e.g. increased oxygen concentration, high concentration of ethanol in the medium or an increase in temperature during cultivation (Gatenholm et al. 2013; Taweecheep et al. 2019a). So far, the reasons for phenotypic variability at the molecular level are not well understood. Coucheron and co-workers pointed to IS1031 insertion elements as the cause of *K. xylinus* production instability, although insertions of these elements were detected only in some of the cellulose non-producing mutants (Coucheron 1991). In other studies, genetic analyses of DNA of Cel− found mutations in genes encoding proteins involved in cellulose biosynthesis pathway as the possible reasons for disappearance of BNC production ability (Table 3). The cellulose negative phenotype may spontaneously appear as well as be reversed. It has often been reported that after several passages of Cel− forms in a static culture, it is possible to restore BNC biosynthesis ability (Matsutani et al. 2015; Taweecheep et al. 2019b). Genomic DNA analyses of such cells (called ‘revertants’) have identified point mutations (insertions or deletions) in the *bcsB* and *bcsC* genes which caused the frameshifts restoring the BcsB or BcsC activity. It is interesting that the point mutations in *bcsCI* not only resulted in the restoration of the BNC biosynthesis capability but also caused up to 6.5-fold increase in BNC yield (Taweecheep et al. 2019b). Moreover, the same study has shown that a single mutation in the *bcsCI* gene may not only improve BNC biosynthesis but also lead to changes in its structure and properties. The authors received two converters (R37-4 and R37-9) synthesising BNC of 2-fold higher fibre density and improved mechanical properties, in comparison to the parental strain.

**Table 3:** Effect of mutation on BNC biosynthesis and/or BNC properties

Strain			Disrupted gene or a fragment	Mutation		Mutation conditions or type of mutagenesis	Phenotypic effects	References
**Culture conditions or environment effect on the formation of cellulose-non-producing (Cel−) forms**								
*K. xylinus* E25	Cel−		Fragment downstream of ORF encoding phosphoglucomutase	A single nucleotide (T) deletion. Inhibited expression of phosphoglucomutase and glucose-1-phosphate uridylyltransferase		Repeated 3 times passages under agitated culture conditions (SH medium, 48 h, 30 °C, 90 rpm) of the parental strain	No BNC production	(Krystynowicz et al. 2005)
*K. europaeus*	LMG 18494		*bcsCI* (cellulose synthase subunit CI)	Large deletion (1900-bp deletion)		Isolation from vinegar	No BNC production	(Andrés-Barrao et al. 2011)
	LMG 18890		*bcsCI*	5-bp deletion				
*Komagataeibacter medellinensis*	NBRC 3288 (parental strain)		*bcsBI* (cellulose synthase subunit BI)	17-bp deletion	Frameshift. Gene disruption by stop codon introduction	Isolation from vinegar	No BNC production	(Ogino et al. 2011)
	R1	Revertants			^a^1-bp (C) deletion at the 277486th position of the gene. Frameshift and BcsBI restoration	Repeated static culture of the parental strain	Restoration of BNC production	(Matsutani et al. 2015)
	R2				^a^1-bp (C) deletion at the 277491th position of the gene, frameshift and BcsBI restoration			
*K. oboediens*	MSKU 3 (wild type)		*bcsCI* (cellulose synthase subunit CI)	–		Isolated from rotten fruit samples in Thailand; thermotolerant strain (growth up to 39 °C)	BNC yield of 0.33 g/L. BNC fibril diameter size: 70.52 nm; density: 0.43 ± 0.05 g cm^−3^. Mechanical properties of BNC; tensile strength: 73.94 ± 16.94 MPa, Young’s modulus: 5.83 ± 0.69	(Taweecheep et al. 2019a, b)
	E3 (parental strain for revertants)			^b^A single nucleotide (T) insertion at the 2135th position of *bcsCI*	Frameshift. Gene disruption by stop codon	Repeated static cultivation of MSKU 3 strain for 63 passages (YPGD1A medium containing increasing concentration of ethanol)	No BNC production. High acetic acid production ability	
	R30-3	Revertants			2-bp (GC) insertion at the 2145th position. Frameshift and two amino acid alterations (N713E and Q714P) and one amino acid addition (A715)	Repeated static cultivation of E3 strain (YPGD1A3E medium)	Increased BNC yield **(**2.15 g/L). BNC fibril diameter size: 65.9 nm; density 0.42 ± 0.02 g cm^−3^. Reduced mechanical properties of BNC (ca. 2-fold ↓); tensile strength: 43.56 ± 10.98 MPa, Young’s modulus: 3.60 ± 0.40	
	R30-12				1-bp (C) deletion at the 2149th position. Frameshift and four amino acid substitution (N713E, Q714P, R715A and G716W)	Repeated static cultivation of E3 strain (YPGD medium)	Increased BNC yield (0.53 g/L). BNC fibril diameter size: 70.14 nm; density: 0.54 ± 0.01 g cm^−3^. Mechanical properties of BNC; tensile strength: 73.94 ± MPa; Young’s modulus: 5.14 ± 0.58	
	R37-4				1-bp (T) deletion at the 2132nd position. Frameshift and a single amino acid substitution (L711R)	Repeated static cultivation of E3 strain (YPGD medium)	Increased BNC yield (1.12 g/L). BNC fibrils diameter size: 59.14 nm; density: 0.72 ± 0.05 g cm^−3^. Increased mechanical properties of BNC (ca. 2-fold ↑); tensile strength: 158.72 ± 28.29 MPa; Young’s modulus: 8.75 ± 1.54	
	R37-9				1-bp (A) deletion at the 2139th position. Frameshift and a single amino acid substitution (N713D)		Increased BNC yield (0.7 g/L). BNC fibril diameter size: 34.58 nm; increased density (2-fold ↑): 0.85 ± 0.07 g cm^−3^. Increased mechanical properties of BNC (2-fold ↑); tensile strength: 159.47 ± 29.76 MPa; Young’s modulus: 9.83 ± 0.69	
**Transposon mutagenesis**								
*K. hansenii* ATCC 23769	5		*acsA* (cellulose synthase subunit A)	Insertion site 1893 bp, gene disruption		Tn 5 transposon insertion mutagenesis	No or reduced BNC production	(Deng et al. 2013)
	10		*acsC* (cellulose synthase subunit C)	Insertion site 1984 bp, gene disruption				
	I-13		*ccpAx* (cellulose complementing protein *Acetobacter xylinum*)	Insertion site 40 bp, gene disruption				
	I-7		*dgc1* (diguanylate cyclase)	Insertion site 656 bp, gene disruption				
	V-31		*dgc1* (diguanylate cyclase)	Insertion site 242 bp, gene disruption				
	II-23		*crp− fnr* (transcriptional regulator, Crp/Fnr family protein)	Insertion site 187 bp, gene disruption				
*K. hansenii* ATCC 23769	I-23		*AlaR* (alanine racemase)	Insertion site 628 bp, gene disruption		Tn 5 transposon insertion mutagenesis	Reduced BNC production and crystallinity	(Deng et al. 2015)
	#52		*LDC* (lysine decarboxylase)	Insertion site 347 bp, gene disruption				
**Mutagenisation by chemical and/or physical agents**								
*K. xylinus* ATCC 53582	plr 15		*bcsA* (cellulose synthase subunit AI)	Substitution of nucleotide G to A at position 1345 bp of the gene; A449T substitution in the BcsA (missense mutation)		EMS (ethyl methane sulfonate) mutagenesis and screening on a medium containing10 μM pellicin	Increased BNC synthesis rate; reduced crystallinity; resistance to pellicin	(Salgado et al. 2019)
*K. hansenii*	HDM 1–3 (wild type)		–	–		Isolated from rotten *Actinida chinesis* Planch	BNC yield of 1.43 g/L	(Li et al. 2016)
	Br-12		Three specific fragments were identified: TonB-dependent transport (TBDT, 779 bp, LCL 80534); exopolysaccharides output protein (PePr, 162 bp, LCL77813); unknown protein (380 bp, LCL57743)	AFLP-based analysis between mutant lines and the wild type		DES (diethyl sulphate) mutagenesis	Decreased BNC yield (0.56 g/L). Increased gluconic acid production (67.53%)	
	Br-3		nd			DES (diethyl sulphate) mutagenesis and screened and selection of mutant in a medium containing NaBr–NaBrO_3_	Increased BNC yield (2.45 g/L). Decreased gluconic acid production (10.23%)	
	Co-5		Two specific fragments were identified: *acsD* and *galE* (UDP-galactose-4-epimerase)			Mutagenesis of the Br-3 mutant by ^60^Co-γ radiation and screened and selection of mutant in medium containing NaBr–NaBrO_3_	Increased BNC yield (3.38 g/L). Decreased gluconic acid production (54.79%)	

YPGD medium (0.5% of glucose, 1% of yeast extract, 1% of polypeptone and 2% of glycerol) containing 1% acetic acid and ethanol (YPGD1A3E)

^a^All the mutations were found to occur in a small C-rich region (CCCGGCCC) in *bcsBI*

^b^All the mutations were found to occur in a small region (TGCTGAACCAGCGTGGC) in *bcsCI*, which seems to be a sensitive region for spontaneous mutation

Mutagenisation with the use of transposons was another approach used to identify genes influencing BNC biosynthesis (Table 3). This type of mutagenisation was applied by Deng et al. and showed that the yield and crystallinity of BNC are influenced by the *alaR* and *LDC* genes encoding alanine racemase and lysine decarboxylase, respectively (Deng et al. 2015). AlaR and LDC proteins are required for maintaining of peptidoglycan integrity. Mutants with interrupted *alaR* and *LDC* genes synthesised less BNC, which was less crystalline, unevenly distributed, with some regions appearing to contain non-cellulose polysaccharides. Ion chromatography–based analysis showed an increase in the number of monosaccharides (galactose and mannose) associated with non-cellulose polysaccharides in comparison to the parental strain. It would be interesting to further investigate the influence of overexpression of these genes on BNC synthesis and properties.

Another strategy to improve cellulose biosynthesis can be the use of mutagenisation with chemical and/or physical agents (Table 3). Culture acidification due to organic acid production is a known factor limiting growth and BNC biosynthesis by the *Komagataeibacter* species (De Wulf et al. 1996). Based on these observations, Li and co-workers aimed to select a high-yield BNC producer by screening for low acid yielding mutants using a combined mutagenesis (treatment with diethyl sulphate coupled with ^60^Co-γ irradiation and proton suicide on medium containing NaBr–NaBrO_3_) (Li et al. 2016). Two mutants were obtained during this study (Br-3 and a Co-5) which synthesised more BNC (1.5 and 2 times more, respectively) and less gluconic acid (10% and 55% less, respectively) in comparison to the wild-type strain. Moreover, the study generated another mutant, Br-12, which synthesised 61% less BNC and 68% more gluconic acid. By employing amplified fragment length polymorphism (AFLP) analysis, the authors explored the genetic differences between the mutants and the wild type strain (see Table 3). The same authors investigated further the physiological basis of acid stress resistance in *K. hansenii* HDM1-3 and showed that it is associated with fatty acids variation in the cell membrane (Li et al. 2019). The team observed changes in the expression of genes of fatty acid dehydrogenase (*des*) and cyclopropane synthase (*cfa*) which encode the key enzymes for the synthesis of unsaturated fatty acids (namely, oleic acid (C18: lw9c) and cyclopropane fatty acid (C19-cyc)). The results of this study encourage further exploration and the manipulation of fatty acids biosynthetic pathway and propose a novel strategy to improve the efficiency of BNC production by the strains of the *Komagataeibacter* genus.

### Molecular and synthetic biology tools for targeted strain manipulation

Until recently, targeted genetic modifications of *Komagataeibacter* spp. were uncommon and limited to the use of two expression vector backbones, namely pSA (Tonouchi et al. 1994; Nakai et al. 1999) and, more frequently, pBBR122 (Setyawati et al. 2007, 2009; Yadav et al. 2010; Florea et al. 2016; Liu et al. 2018b). Induction of gene disruption through a homologous recombination was commonly performed with the use of non-replicating plasmid disruption cassette carriers, such as pUC19, pT7Blue, pET14 or pHSG399 (Table 4). At present, one can expect intensification of genetic engineering of different *Komagataeibacter* strains due to the progress in the application of three new vector backbones for endogenous and heterologous gene expression, namely pTI99, pTSa and pSEVA331Bb (Fang et al. 2015; Florea et al. 2016; Jacek et al. 2018, 2019b; Gwon et al. 2019). Moreover, the performance of various promoters, ribosome binding sites (RBS) and terminators has been characterised in the first two studies applying synthetic biology tools in *Komagataeibacter* hosts (Florea et al. 2016; Teh et al. 2019). These studies together give a comprehensive guide for the construction of vectors of a high potential to be effective in the numerous species from this genus. These recent achievements include the use of quorum sensing (QS) synthetic biology elements known to be functional in *E. coli* (Florea et al. 2016; Walker et al. 2018). The first example of employment of these elements was the application of inducible *lux* promoter and *luxR* gene for modification of *K. rhaeticus* strain (Florea et al. 2016). This study showed that the capability of cellulose production was lowered and eventually switched off with increasing concentration of *N*-acyl homoserine lactone (AHL) in the media. Another study reported the construction of Sender and Receiver recombinant *K. rhaeticus* strains (Walker et al. 2018). The first strain produced AHL in response to an environmental signal, while the second strain induced recombinant protein expression (red fluorescent protein, RFP) in response to AHL in a concentration-dependent manner. The most recent synthetic biology study was published by Teh et al. and consists of a comprehensive review of performance of several promoters, RBS and terminator sequences tested in the pSEVA plasmid backbone in three *Komagataeibacter* strains (*K. hansenii* ATCC 53582, *K. rhaeticus* iGEM and *K. xylinus* ATCC 700178). Importantly, the first use of CRISPR interference (CRISPRi), targeting chromosome-encoded *acs* operon in *K. hansenii* ATCC 53582, was described in this study (Teh et al. 2019). Summarising, this work paves the way for studies aiming at obtaining of self-immobilised *Komagataeibacter* hosts expressing biological circuits of desired function and which may be applied as, e.g., biosensors in intelligent packaging material.

**Table 4:** Genetic engineering tools used in various *Komagataeibacter* strains

Induction of gene expression				
**Vector backbone**	**Promoters**	**Derived constructs**	***Komagataeibacter*strain**	**References**
pSA19	lac	pSA-SD pSA-SD-S11E	*K. sucrofermentans* BPR 2001	(Nakai et al. 1999)
pBBR122	Bla lac	pBla-Vhb-122 pLacDAAO-122 pBla-Vhb-122 pBBR-Glc-NAc	*K. xylinus* BCRC 12334 *K. xylinus* BCRC 12334 *K. xylinus* CGMCC 2955 *K. xylinus* 10245	(Setyawati et al. 2007) (Setyawati et al. 2009) (Liu et al. 2018b) (Yadav et al. 2010)
pTI99	trc	pTI99-cdrS pTI99-motAB −motA; −motB	*K. hansenii* ATCC 23769 *K. hansenii* ATCC 23769 *K. hansenii* ATCC 53582	(Fang et al. 2015) (Jacek et al. 2018) (Jacek et al. 2018)
pTSa	tac gapA	pTSaEX1 −pfk −pgi; −pgk; −fba; −tpi; −gap; −gpm; −pck; −mae; pTSaEX2	*K xylinus* DSM 2325	(Gwon et al. 2019)
pIN01	tac	pIN01-crp	*K xylinus* DSM 2325	(Gwon et al. 2019)
pSEVA331Bb	J23104 lux tet Bad J23100 J23101 J23102 J23104 J23105 J23108 J23109 J23110 J23115 J23119 J23119-A27T	J-sRNA-331Bb pLux01 pLux02 pTet01 pTet02 pReceiver pSender pSEVA331Bb-mRFP pSEVA331Bb-NAG5-AGM1-UAP1	*K. rhaeticus* iGEM *K. rhaeticus* iGEM *K. rhaeticus* iGEM *K. hansenii* ATCC 53582 *K. xylinus* ATCC 700178	(Florea et al. 2016) ^a^ (Walker et al. 2018) (Teh et al. 2019)
pLBT	Promoter-less miniTn10	pLBT::lacZ:kan	*K. xylinus* ITDI	(Battad-Bernardo et al. 2004)
**Knock-out or inhibition of gene expression**				
**Vector backbone**	**Method/selection marker**	**Obtained constructs**	***Komagataeibacter*strain**	**References**
pUC18	HR/*amp*^*R*^ HR/*kan*^*R*^	pUCA-EP	*K. sucrofermentans* BPR 2001	(Ishida et al. 2002)
		acsD deletion	*K. hansenii* ATCC 23769	(Hu et al. 2010)
pUC19	HR/*amp*^*R*^	T-GDH-Amp	*K. xylinus* BCRC 12334	(Kuo et al. 2015)
pT7Blue	HR/*cat-1*	pT7-Blue-PGDHC-I	*K. sucrofermentans* BPR 2001	(Shigematsu et al. 2005)
pET14B	HR/*kan*^*R*^	motA motB and motAB disruption	*K. hansenii* ATCC 53582	(Jacek et al. 2019b)
pHSG399	HR *kan*^*R*^ HR *amp*^*R*^	Dgc1 cmcAx pSA-ORF2/k (cpcAx deletion)	*K. sucrofermentans* BPR 2001	(Bae et al. 2004) (Nakai et al. 2013) (Nakai et al. 2002)
pSEVA331Bb	sRNA	J-sRNA-331Bb (co-expression of Hfq and sRNA targeting UGPase mRNA)	*K. rhaeticus* iGEM	(Florea et al. 2016)
	ter	ECK120033736 ECK120010818 ECK120010799 ECK120051401 BBa_B0010 L3S2P21 L3S3P21 L3S2P24 L3S2P44 L3S1P47	*K. rhaeticus* iGEM *K. hansenii* ATCC 53582 *K. xylinus* ATCC 700178	(Teh et al. 2019)
	Protein degradation tags	LAA AAV LVA DAS DAS + 2	*K. rhaeticus* iGEM *K. hansenii* ATCC 53582 *K. xylinus* ATCC 700178	
	CRISPRi	pSEVA331Bb-Cas9-3xFLAG-sgRNA	*K. hansenii* ATCC 53582	

*HR* homologous recombination, *CRISPRi* deactivated Caspase9-based system = CRISPR interference, *sRNA* short RNA mediated, *ter* translation terminator, *kan*^*R*^ kanamycin resistance gene, *cat-1* chloramphenicol acetyltransferase gene, *amp*^*R*^ ampicillin resistance gene

^a^Other vector backbones reported here as replicating in *K. rhaeticus* iGEM but not studied further: pBla-Vhb-122; pSEVA321Bb, pBAV1K-T5-sfGFP

All the vectors used for gene overexpression in the *Komagataeibacter* strains together with vectors used in gene disruption and deletion approaches are summarised in Table 4. In most cases, the functionality of the constructs was verified in a limited number of strains and their compatibility with other *Komagataeibacter* species has yet to be tested.

### Improvement in cellulose productivity

Genetic modification of the *Komagataeibacter* strains has been used to generate a few industrially relevant mutant strains with improved cellulose productivity. These improvements have been achieved either through metabolic pathway modulation (Shigematsu et al. 2005; Kuo et al. 2015; Gwon et al. 2019) or heterologous gene expression (Setyawati et al. 2007; Liu et al. 2018b; Jang et al. 2019).

From a cellulose productivity point of view, a desirable direction of metabolic shift is limitation of production the major side-product from glucose which is gluconic acid. This goal was reached, e.g. via deactivation of a gene encoding membrane-bound glucose dehydrogenase (GDH; Shigematsu et al. 2005; Kuo et al. 2015). Active oxidation of glucose into gluconic acid has two undesired effects in *Komagataeibacter* cultures: pH lowering and quick loss of glucose from medium. These obstacles were overcome in two GDH-deficient mutants: the GD-1 in the *K. xylinus* BPR2001 strain (Shigematsu et al. 2005) and, more recently, the GDH-KO in the *K. xylinus* BCRC 12334 strain (Kuo et al. 2015). Both these strains were obtained by means of homologous recombination (Table 4). The GDH-deficient mutants produced BNC at ca. 2-fold higher yield than the parental strains. What is more, the GD-1 strain was shown to be able to effectively produce BNC on enzymatically saccharified potato pulp (BC yield reached 5.0 g/L without and 7 g/L after ethanol supplementation; Shigematsu et al. 2005).

Another example of a genetically improved cellulose-producing strain was expressing *Vitreoscilla* haemoglobin (VHb) which assured higher ATP synthesis under hypoxic conditions (static culture or lowered O_2_ tension in a closed bioreactor) (Setyawati et al. 2007; Liu et al. 2018b). In both studies, the observed increase of cellulose production yield by the VHb+ strains was in the range of 1.5- to 1.7-fold.

A more recent study involving *K. xylinus* DSM 2325 strain has shown that the reduction of gluconic acid production can be achieved through manipulation of regulatory proteins, besides the direct catabolic enzymes (Gwon et al. 2019). This study generated a strain with reduced gluconic acid synthesis yield (39.2% in the mutant and 64.8% of glucose in the parental strain) after introduction of an additional copy of the *crp* (Crp/Fnr) gene, expressed from a plasmid, under the *tac* promoter control. Apart from *crp* overexpression, the mutant strain (named S.Koma-pfkA/crp) used by Gwon et al. harboured the *pfkA* gene fused in chromosome (Gwon et al. 2019). Selection of this gene was based on the analysis of the aforementioned GEM for this strain. The authors speculate that this change caused redirection of metabolic flux to the Embden–Meyerhof–Parnas (EMP) pathway, which was predicted to be inactive in their parental strain, enabling higher ATP production and, as a result, an increased BNC production yield (4.3 g/L in mutant; 3.5 g/L in parental strain). Constitutive expression of several other genes from EMP pathway (namely *pgi*, *pgk*, *fba*, *tpi*, *gap*, *gpm*, *pck* and *mae*) was also tested but did not influence the final BNC yield. The finally optimised strain expressing *pfkA* and *crp* genes (S.Koma-pfkA/crp) gave 4.5 g/L cellulose production rate and was used for batch fermentation in a 30-L bioreactor. After optimisation of fermentation process, the authors obtained 3.3 times higher cellulose production yield by a mutant strain related to the parental one.

When discussing genetic modification achievements in the field of BNC production, one should not overlook the early constructions of strains capable of carbon assimilation from sucrose (Tonouchi et al. 1998a, 1998b; Nakai et al. 1999) or lactose (Battad-Bernardo et al. 2004), both being more economical carbon sources than glucose. Particularly an efficient sucrose-assimilating mutant strain, which showed 2–3-fold increase in cellulose productivity, when compared to the parental strain, was obtained by expression of mung bean sucrose synthase on the pSA plasmid (Table 4) (Nakai et al. 1999). The lactose-assimilating strain (ITz3) harboured transposon-mediated chromosome integration of *E. coli lacZ* gene (Battad-Bernardo et al. 2004). The ITz3 mutant strain differed from *K. xylinus* ITDI parental strain by its ability to produce cellulose on media containing lactose and on whey, which is the most common by-product of the dairy industry. The ITz3 strain constitutively expressed enzymatically active beta-galactosidase and achieved BNC synthesis yield from 0.73 g/L on whey up to 1.30 g/L on the PYSL4 medium, even though the parental strain was unable to produce cellulosic pellicle in any of these media.

### Modification of BNC structure

Having regard to the diversity and still expanding scopes of BNC applications, it is still essential to take full advantage of its exceptional structure and properties. The development of novel BNC-based materials with unique properties has been the subject of an extensive research over the last two decades. Numerous modification methods have been explored to inquire BNCs with novel or improved functionalities (Hu et al. 2014). Biosynthetic (in situ) or physical/chemical (ex situ) modifications of cellulose membranes constitute the vast majority of all the explored functionalising methods. Genetic engineering tools, however, offer the possibility of introducing non-invasive changes to cellulose, without the risk of unintentionally influencing its native properties.

**Fig. 2:**
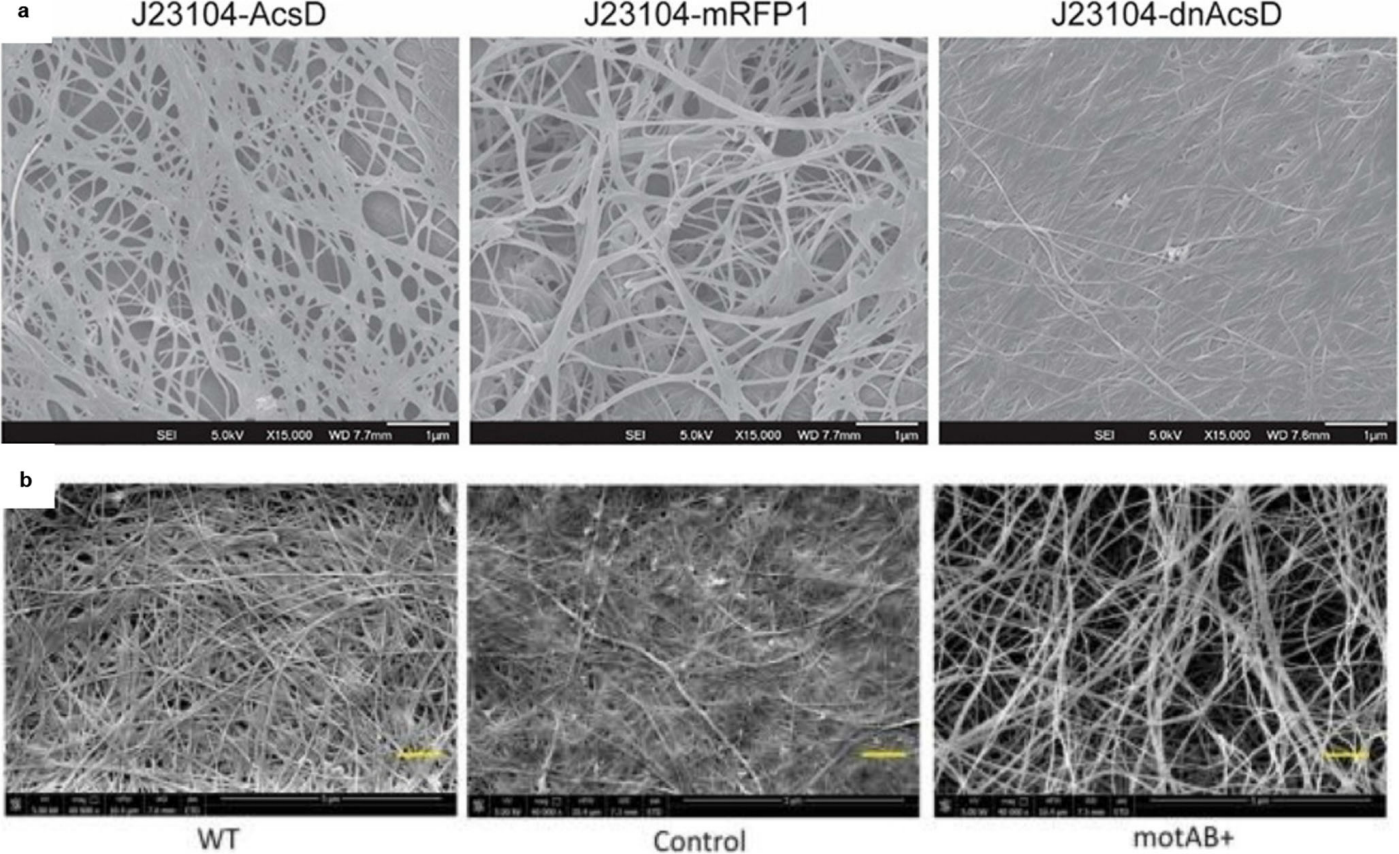
Genetically induced structural changes in BNC network. **a** A dense cellulose matrix of thin fibres produced by the dominant negative AcsD (dnAcsD) *K. hansenii* ATCC 53582 mutant (AcsD—cellulose synthase subunit D; strain expressing wild-type AcsD (J23104-AcsD) or mRFP1 (J23104-mRFP1)). Reprinted with permission from *ACS Synth. Biol.* (2019, 8, 4, 708–723). Copyright (2019) American Chemical Society. **b** A loose BNC network produced by *K. hansenii* ATCC 23769 mutants overexpressing the motility-related genes *motA* and *motB* (WT—wild-type strain, Control—strain transformed with an empty vector; motAB+—mutant with *motA* and *motB* overexpression). Reprinted from Jacek et al. (2018)

Cellulose, as a highly porous, nanofibrillated material, has been the subject of numerous structural modifications. Up to date, only a very few of them employ recombinant strains, what result from still ongoing research on the role of particular genes involved in metabolism of cellulose synthesis. The main target genes are the constituents of bacterial cellulose synthase (BCS) operon. Teh and co-workers revealed that the dominant negative AcsD (dnAcsD) expression causes the production of a dense cellulose matrix (Fig. 2a; Teh et al. 2019). At the same time, dnAcsD-overexpressing cells secreted thinner fibres than those produced by the wild-type and other bacterial strains tested in the study. It was speculated that the presence of dnAcsD might disrupt the crystallisation of thicker subelementary fibrils after glucan chain extrusion. Such results suggest it might be possible to tailor the structure of BNC by tuning the ratio of wild-type AcsD to dnAcsD.

Going beyond the operon coding for cellulose synthase, since more and more genomes of cellulose-producing strains are published and analysed, the scientists have been investigating the roles of genes responsible for general cellular behaviour. One example of such approach is the study on the *motA* and *motB* genes which build up a proton pump and which are probably involved in cell motility (Jacek et al. 2018, 2019b, 2019c). Jacek et al. subjected *K. hansenii* ATCC 53582 to disruption and complementation of one or both of the mentioned genes, whereas in *K. hansenii* ATCC 23769 induced *motA* and *motB* genes overexpression. The latter mutation caused bacterial cell elongation (or formation of filaments) and intensification of colony-spreading ability. Microscopic analysis of the BNC membrane has shown a significant loosening of intra-membrane structure and fibre thickening was observed (Fig. 2b; Jacek et al. 2018).The opposite effect was observed in the *motA−* and *motB−* disruption mutants which were synthesising more dense and compact cellulose (Jacek et al. 2019b). Yet, both the examples indicate that a change in motility-related genes indirectly influences the nanostructure of cellulose, making it more porous and relaxed when the recombinant cells are elongated, and their movement is intensified. Moreover, the mutant-derived BNC was proved to preserve biocompatibility and chemical properties of BNC of the parental, non-modified strains which still makes it highly valuable in medical applications.

### Synthesis of BNC nanocomposites

**Fig. 3:**
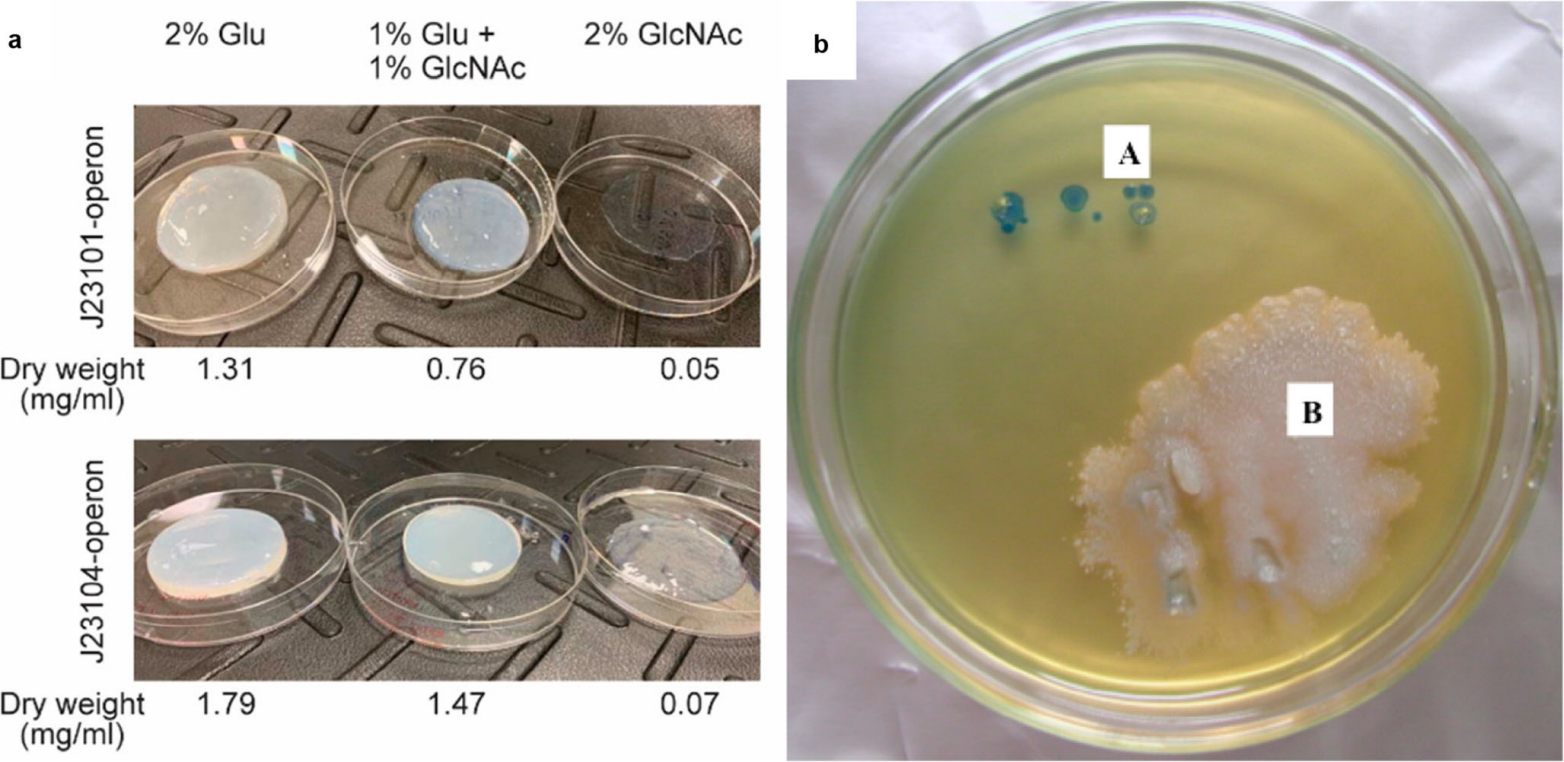
Examples of genetically engineered BNC bionanocomposites. **a** Images showing the cellulose–chitin copolymer produced under two different promotors, using different concentrations of glucose (Glu) or GlcNAc. Reprinted with permission from *ACS Synth. Biol.* (2019, 8, 4, 708–723). Copyright (2019) American Chemical Society. **b** Curdlan–cellulose composite produced by genetically engineered *K. hansenii* AY201 visualised by aniline blue staining (**a**—colonies of the mutant strain expressing curdlan synthase; **b**—colonies of the control strain). Reprinted from *Biomacromolecules* (2015, 16, 10, 3154–3160)

Last but not least, genetic manipulation has been shown to be an attractive approach for producing BNC nanocomposites. Yadav et al. were the first to engineer *K. xylinus* to rationally redesign the flow of cellular metabolites so as to incorporate *N*-acetylglucosamine (GlcNAc) sugar residues into glucan chains during the biosynthesis (Yadav et al. 2010). The authors took the advantage of the fact that *K. xylinus* (formerly *Ga. xylinus*) can utilise both UDP-glucose and UDP-GlcNAc as substrates. In order to produce a composite of bacterial cellulose and chitin, a recombinant strain of *K. xylinus* 10245 was developed. An operon composed of three genes from *Candida albicans* (AGM1, NAG5 and UAP1) responsible for UDP-GlcNAc synthesis was expressed under the control of *bla* promoter in *K. xylinus* (Table 4). The modified strain was able to produce activated cytoplasmic UDP-GlcNAc monomers accessible to cellulose synthase to join both glucose and GlcNAc, and synthesise a chimeric polymer. The generated material contained over 18-fold more GlcNAc, as compared to the control BNC; it was more facile to enzyme degradation and displayed improved degradability in vivo. The same concept of de novo synthesis of chitin–cellulose composite was adopted by Teh et al. in order to demonstrate the utility of a genetic toolkit for synthetic biology applications in *Acetobacteraceae* family (Teh et al. 2019). The three genes responsible for UDP-GlcNAc synthesis were expressed in the wild-type *K. xylinus* ATCC 700178 under either a weak (J23101) or a strong promoter (J23104) revealing that more cellulose is formed in the latter case. Nevertheless, both systems successfully generated a composite material which synthesis level and quality strongly depends on the content of the both substrates (glucose and GlcNAc) in the medium (Fig. 3a).

In 2015, the group of Professor Kondo developed in vivo a curdlan–cellulose bionanocomposite by applying a genetically modified *K. hansenii* AY201 (*Ga. xylinus* in the original work; Fang et al. 2015). Curdlan is an exopolysaccharide known in biomedical applications because of its nonionic gelation properties and low toxicity. Genetically engineered strain was obtained by a direct modification of cellulose-synthesising metabolic pathway of bacteria to secrete a mixture of cellulose and curdlan (β-1,3-glucan). Similarly, as during cellulose biosynthesis, curdlan synthesising microorganisms use UDP-glucose as a precursor for its production. To introduce a curdlan biosynthesis system into *K. xylinus*, the authors expressed curdlan synthase (*crdS*) gene from the most efficient producer of this biopolymer, *Agrobacterium* sp. ATCC 31749. The other accessory genes of curdlan excretion were not included in order to provide the exclusive extracellular secretion of curdlan–cellulose nanocomposite directly from a bacterial cell. The resulting strain was shown to simultaneously secrete curdlan and crystalline cellulose nanofibres (see Fig. 3b), providing a composite material of slightly changed surface morphology (curdlan to some extent covered the pores of cellulose) but still with preserved crystallinity. It is believed that the constantly growing state of knowledge of function of *Komagataeibacter* genes will enable further research on efficient engineering of these bacteria for biomanufacturing of novel cellulose-based materials and numerous commercially valuable products.

## Conclusions and future perspectives

This review aimed to discuss the current progress in the systemic understanding of *Komagataeibacter* physiology. Gaining of this perspective depends on the availability of high-quality omics datasets which improve genome functional annotation. This information is crucial for building of robust genome-scale metabolic models and selection of new targets for genetic engineering. Targeted and untargeted approaches for strain improvement have shown that it is important to investigate genes indirectly related to metabolic pathway of BNC synthesis as well as transcriptional regulators. Moreover, low immunogenicity and good biocompatibility of BNC products is maintained when using recombinant bacteria instead of chemical actions on this material which is especially important in biomedical field. Preserving its native physico-chemical properties, cellulose produced by modified microorganisms may display altered 3D nanostructure, porosity or density. On the other hand, the importance and potential of bacterial nanocellulose may be greatly increased when applying genetically engineered strains in combination with chemical and physical modifications of BNC. The intentional interconnections between specific metabolic pathways in the *Komagataeibacter* might result in the design of novel, chimeric biopolymers. It seems inevitable that still growing knowledge in the field of genome-dependent metabolic mechanisms influencing the process of BNC biosynthesis will be constantly widening the scope of its possible targeted modifications, as well as its final implementation.
